# Indoor and outdoor human behavior and myopia: an objective and dynamic study

**DOI:** 10.3389/fmed.2023.1270454

**Published:** 2023-10-31

**Authors:** Elise N. Harb, Elsie Shin Sawai, Christine F. Wildsoet

**Affiliations:** Herbert Wertheim School of Optometry and Vision Science, University of California at Berkeley, Berkeley, CA, United States

**Keywords:** myopia, wearable technologies, visual environment, outdoor time, near work

## Abstract

**Significance:**

Myopia holds significant public health concern given its social, ocular disease and economic burdens. Although environmental factors are primarily to blame for the rapid rise in prevalence, key risk factors remain unresolved.

**Purpose:**

The aim of this study was to objectively characterize, using a wearable technology, the temporal indoor and outdoor behavioral patterns and associated environmental lighting characteristics of young myopic and nonmyopic University students.

**Methods:**

Participants were recruited to continuously wear an Actiwatch for 3 weeks, during either or both academic and non-academic periods. The device allows continuous recording of activity and incident light. Recorded illuminance levels were used as a proxy for outdoors (>1,000 lux), with the dynamics (interval frequency and duration) of indoor and outdoor activities, as well as lighting characteristics derived. In addition, participant input regarding near work was obtained daily. Participants were classified by both myopia and axial length status (based on collected refractive error and biometry data) for the purpose of data analysis.

**Result:**

A total of 55 students, aged 18 to 25 years of age, participated. Overall, the dosing of indoor and outdoor activities was similar across participants, regardless of myopia status, during the academic period. Nonetheless, an apparent difference in the timing of outdoor activities was noted with myopes going outdoors later in the day, particularly during the weekend (*p* = 0.03). While a trend was observed between increased lighting levels experienced outdoors and shorter axial lengths, there was no significant relationship with myopia status. Noteworthy, participants generally significantly overestimated time spent outdoors, compared to Actiwatch-derived estimates of the same.

**Conclusion:**

While the findings from this cohort of young adult students did not reveal substantial myopia-related differences in behavior, the power of a more objective and dynamic approach to quantifying behavior cannot be understated, providing argument for general adoption of wearable technologies in future clinical myopia studies.

## 1. Introduction

The apparent protective effect of increased outdoor exposure against myopia development and progression continues to draw significant attention [for a review see Xiong et al. (1)]. However, while exposure to high light intensity, as encountered outdoors, has been the focus of attention of several related studies (2–4), no clear consensus has been reached. To-date, four randomized control trials investigating the potential therapeutic benefit of increased school recess time have been completed, all in East Asia (China and Taiwan), where the myopia prevalence figures are among the highest worldwide (5–8). While reported treatment effects varied across these studies, they were generally small, providing no direct evidence for a therapeutic benefit of bright light. (8) Moreover, little attention was paid to the many other differences that exist between indoor versus outdoor visual environments and experiences, despite evidence from animal models of the important influences of both visual spatial and temporal factors as determinants of optical defocus effects on early eye growth (9, 10). While studies aimed at modeling and/or quantifying retinal image characteristics corresponding with representative indoor and outdoor environments (11, 12), point to substantial differences, such information is lacking in studies of “habitual” indoor and outdoor visual environments in the context of human myopia.

Another significant deficiency in most studies attempting to quantify visual experiences in myopia-related studies has been the reliance on subjective reports captured through questionnaires, which are well recognized to be inaccurate (13, 14) and limited in their ability to capture potentially critical details, such as the temporal pattern of exposure (dosing), which has proven influential in modulating eye growth in animal models (15–17). Related to indoor visual environments, simple metrics, such as how many books read by a child, as derived from subjective reports on the amount of near work undertaken by a child, have shed little light on the role of the latter in myopia development and/or progression. For example, a meta-analysis investigating the association between hours of near work and the presence of myopia found that while increased near work was associated with myopia, the effect was small and not clinically meaningful (18). Nonetheless, a recent study pointed to a strong link between time spent in school and myopia, leading the authors to conclude that the intensity of schooling was an important driver of myopic shifts in refraction (19).

Given advances in wearable electronic technologies, the opportunity to collect objective and thus more reliable (less biased) human behavior data presents itself, with the potential to reveal yet undiscovered myopia-genic aspects of the visual environment and/or human behaviors, not captured in questionnaires. Towards constructing more detailed, objective pictures of outdoor exposure and near work, already published studies have made use of light meters (4, 8, 13, 14, 20), accelerometers (4, 21) and/or spectacle mounted range finders (22, 23), often in conjunction with traditional questionnaires. However, few studies employing such technologies have capitalized on their power to capture the dynamic aspects of light exposures and behavior. Towards correcting such deficiencies, this study aimed to objectively characterize the temporal behavioral patterns of myopic and non-myopic young university students, including the time of day, frequency, and duration of each indoor/outdoor activity interval). To this end, Actiwatches which have light- and motion-sensing capability, were used to capture the lighting characteristics in both indoor and outdoor environments as well as the corresponding activity data for the wearers, which were used to generate temporal profiles of the same. Our hypothesis is that the dynamics of habitual indoor and outdoor activities will be different for myopic compared to non-myopic participants.

## 2. Methods

Young adult students (undergraduate and graduate students on the UC Berkeley campus) were recruited to participate in this study. Exclusion criteria were limited to previous histories of myopia control interventions, eye disease, or eye surgery. This research was reviewed by an independent ethical review board and conforms with the principles and applicable guidelines for the protection of human subjects in biomedical research. To investigate the possible differences in activities during academic (AP) and non-academic (NAP) periods, participants were asked to participate in two measurement sessions, one in each of these periods respectively, separated by approximately 4–6 months. This timing also allowed us to evaluate myopia progression in participants and potential association with behavior differences.

Each measurement session involved two visits (details provided below), at the beginning and end of a two-week observation period over which participants were asked to continuously wear an Actiwatch (Respironics Actiwatch Spectrum Pro). Specifically, participants were instructed to wear the Actiwatch on their non-dominant wrist, over clothes for 24 h a day (except during prolonged swimming), for a 14-day period (including two weekend periods). A built-in off-wrist monitor (watch beeps when not worn) promoted participant adherence to the watch-wearing schedule.

The Actiwatch device continuously measures overall incident illuminance (lux), as well as the spectral composition of light in terms of irradiance (RGB, μW/cm2) (spectral sensitivities; R: 600–700 nm, G: 500–600 nm B:400–500 nm). It also has a built-in accelerometer that measures participant activity (counts per min (cpm)) and sleep/wake periods. Strong between-device correlation of Actiwatch illuminance measures have been reported (24); outputs from this device, which represent averages over 1-min epochs, have also been shown to provide reliable estimates of ambient illuminance (25).

Refractive error and ocular biometric data from the right eyes of participants were collected at beginning of each two-week observation periods. Spherical equivalent refractive errors (SERs) were derived from measurements made under cycloplegia (30 min following 2 drops 1% tropicamide), with an open-field auto-refractor (Grand Seiko WR-5100 K) and axial lengths (AL) were recorded with an IOL Master (Zeiss). For categorical analyzes, myopia was defined as −0.50 D or worse and participants were classified as either myopes or non-myopes, or further classified as non-myopes (NM, *n* = 19), low myopes (LM, −0.50 to −3.25 D, *n* = 16) or moderate to high myopes (HM, < −3.25 D, *n* = 15), based on the definition of myopia and a median split of the SER of the myopic cohort. Similarly, participants were categorized into three AL groups; long (LAL, > 25.05 mm, *n* = 16), medium (MAL, 24.18 to 25.05 mm, *n* = 16) and short (SAL, < 24.18 mm, *n* = 17), based on a tertile split of the cohort.

Analyzes of Actiwatch data were performed off-line, using custom Matlab software, with related data excluded where there was indication that the watch was not worn (as indicated by the off-wrist sensor); incidental coverage of the Actiwatch by clothing was signified by missing data. For each participant, the dynamics of outdoor and indoor exposures were calculated in several ways. In brief, the daily average and variation (SD) in episode frequency and duration, as well as the timing of each episode was calculated. For analysis of the lighting characteristics, the average and variation (SD) in brightness (illuminance, lux) experienced outdoors and indoors, both within an interval (of a given day) and across a day were computed, with daily averages also used to compute two-week averages. The spectral composition of the indoor lighting to which participants were exposed was also characterized in terms of the ratio of long(red):short(blue) wavelength (R:B) irradiances. Data representing weekdays and weekends were separately analyzed.

For comparative purposes, subjective questionnaires were administered to the first 30 participants recruited. The questionnaires inquired about habitual daily outdoor activities during an academic period. Our approach was different from that used in past studies in that: 1) two distinct questionnaires covered weekday and weekend activities, separately and 2) each questionnaire was administered two times, separated by 1 week, where participants were asked to notate their habitual daily time outdoors (in minutes) over the previous week or weekend (depending on which survey was administered). For both weekdays and weekends, averages representing time spent outdoors were calculated from the two related reports provided by each participant and weighted mean daily activity values (in minutes) derived for use in data analyzes.

### 2.1. Outdoor activity analysis

Light intensity consistent with outdoors (≥1,000 lux) was used as a proxy for outdoor activity (4, 14), with three additional qualifiers considered in classifying an interval as ‘outdoor activity’: 1) outdoor activity could only occur during waking hours (as determined by accelerometer data), and 2) outdoor activity for a given day could only occur between regional sunrise and sunset hours (as determined daily by Lawrence Laboratories, UC Berkeley), and 3) small (less than 3 min) spikes in illuminance (>1,000 lux) were not classified as outdoor intervals and likewise, short, small dips (<1,000 lux) in ambient illuminance were ignored in calculating outdoor intervals. Because the spectral composition of sunlight changes across the day (greater contribution of longer wavelengths in the evening) (26, 27), we also calculated the average daily frequency of outdoor exposures occurring before versus after 12 pm for each participant. Lastly, in relation to both myopia and axial length status, we explored potential differences in the temporal patterns of outdoor exposures by generating pooled distributions for the timing of outdoor activities over the entire two-week observation periods.

### 2.2. Indoor activity analysis

Light intensity consistent with indoors (<1,000 lux) was used as a general proxy for indoor activities (4, 14), with several additional factors also considered in defining an interval of ‘indoor activity’: 1) indoor activity could only occur during waking hours (as determined by accelerometer data) and 2) both short duration dips and spikes in ambient illuminance (<1,000 lux, ≥1,000 lux resp.) were ignored in determining indoor interval duration.

### 2.3. Near and intermediate activity analysis

To quantify the near and intermediate activities of our participants over the 2-week observation period, the Actiwatches were programmed to beep nightly, approximately 1 h prior to the reported habitual bedtime of each participant. At this time, participants were required to enter two numerical estimates corresponding to the accumulated hours (between 1 and 15 h), for *that* day spent on: a) near activities, defined as nearer than an arm’s length away, e.g., handheld device use, reading a book, homework, and b) intermediate activities, defined as approximately an arm’s length away, e.g., computer/laptop use, reading music.

From the above Actiwatch near and intermediate activity responses of each participant, their daily averages of total combined near and intermediate work were calculated for both weekdays and weekends. A weighted mean daily activity value was derived from these two reports for each participant for use in comparative data analyzes. Note that in reporting the results from such data analyzes, all near/intermediate activity will be referred to simply as “near” activity.

### 2.4. Statistical analysis

All dynamic and cumulative data were graphically analyzed with respect to ocular metrics, in both continuous and categorical (by AL or myopia group), and comparisons were made between academic and nonacademic periods (AP vs. NAP). Summary statistics are reported in terms of mean ± SD, unless otherwise noted. Differences in behavior related to myopia and axial length status, as well as between academic and nonacademic periods, were explored using as appropriate, unpaired or paired t-tests or ANOVAs were applied using the Excel data analysis toolpak or R-Studio.

## 3. Results

### 3.1. Participants

A total of 55 young adult university students, aged 18 to 25 years of age, participated in this study. The demographics of the participants are summarized in Table 1. Note that the participant cohort was highly unbalanced with respect to gender, with 85% being female. Also of the subset of 15 participants who had data collected during both academic (AP, actively engaged in-person classes) and non-academic (NAP, summer vacation) periods, only four were not myopic. Nonetheless, in relation to their refractive errors, the range of myopia represented was quite wide overall (−0.625 to −8.75 D), with the ranges of SER for the 3 myopia status categories as follows: −3.25 to −8.75 D for the HM group, −1.00 to −3.00 D for the LM group and + 2.375 to −0.375 D for the NM group. Of the 15 participants observed in both AP and NAP periods, none recorded significant myopia progression (range of change in SER: 0 to −0.625 D), possibly reflecting the small sample size and the age of the participants, i.e., young adults. Given the latter finding, no further myopia progression-related analyzes were undertaken. The range of axial lengths for this cohort was also wide, ranging from 21.83 to 27.58 mm, with the corresponding ranges for the three-axial length (AL) categories being 25.05 to 27.58 mm for the high axial length (HAL) group, 24.03 to 24.97 mm for the medium axial length (MAL) group and 21.83 to 23.95 mm for the short axial length (SAL) group.

**Table 1:** Participant characteristics.

Characteristic		Participants (*N* = 55)
Gender	Female Male	*n* = 47 *n* = 8
Age (years)	Range	18–25 y
Sph. Eq. Refractive Error (D)	Mean (±SD)	−2.25 (±2.55)
	Range	2.375 to −8.75
Axial Length (mm)	Mean (±SD)	24.56 (±1.28)
	Range	21.83 to 27.96
Myopes	Myopes (−0.50D or worse) Non-myopes	*n* = 33 *n* = 22
Measured Period (Academic vs. Nonacademic)	Academic (AP) *N* = 49	Myopes = 31
		Non-Myopes = 18
Note: 11 myopes and 4 non-myopes were observed during both AP and NAP	Nonacademic (NAP) *N* = 21	Myopes = 13
		Non-Myopes = 8

In relation to the timing of observations in the study, they were moderately balanced by season, with 36% of the AP cohort being observed in the spring/summer months (March–August), the reminder observed during fall/winter months (September–February). Also not surprisingly, the timing of observations for the subset of participants who were observed in both AP and NAP periods was better counter-balanced, with AP observations made in the fall/winter and NAP observations in the spring/summer for 8 out of the total of 10 participants for whom the timing of observations represented a switch in seasons. Finally, given that the cohort observed in the AP was much larger than that observed in the NAP, only findings for the AP period are described in detail, with comparisons between AP and NAP periods reported only when differences in behaviors were found to be significant.

### 3.2. Outdoor activity

#### 3.2.1. Subjective vs. objective measures

**Figure 1:**
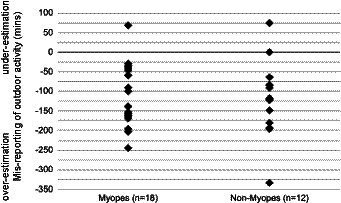
Subjective reports vs. objective recordings of outdoor activity. Both myopes and non-myopes tended to over-estimate time spent outdoors on subjective questionnaires, although myopes to a slightly less extent on average [mean overestimation (SD): myopes: 116 (68.3) mins, non-myopes: 145 (74.2) mins].

For a subset of 30 participants, both subjective and objective measures of outdoor activities were captured during an AP. All but three participants significantly over-estimated by a considerable amount, the time spent outdoors for a given day compared to estimates derived from Actiwatch data (average difference 120 min, *p* < 0.05, Figure 1). This is despite the relatively short time interval between activities and completion of related questionnaires (previous weekend or week), and the compared data representing the average of two sets of data, collected over two consecutive weeks. In relation to myopia status, both myopes and non-myopes over-estimated the time spent in outdoor activities by a similar amount (myopes: 103%, non-myopes: 108%), although the discrepancy in results for the myopic participants tended to be smaller on average compared to those of the non-myopes [mean (SD): 116 (68.3) mins, myopes; 145 (74.2) mins, non-myopes; *p* = 0.29; Figure 1].

#### 3.2.2. Outdoor activity dynamics

**Figure 2:**
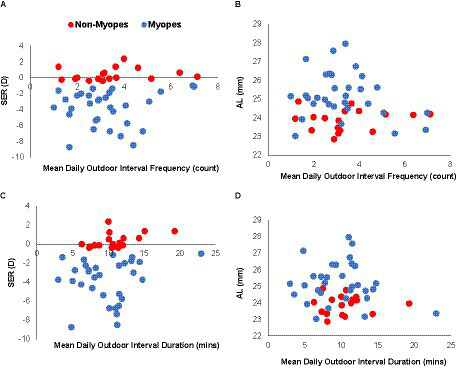
Outdoor activity interval dosing in myopes and non-myopes. Refractive error [SER, **(A,C)**] and axial length [AL, **(B,D)**] plotted against mean frequency of daily outdoor intervals **(A,B)** and interval duration **(C,D)** for myopes (blue) and non-myopes (red); behaviors are similar for the two groups and neither parameter is significantly correlated with either SER **(A,C)** or AL **(B,D)**.

During APs, participants went outdoors infrequently (mean, SD: 3.33 (1.51) intervals/day, range: 1–7 intervals/day) and for only very short periods of time (mean, SD interval duration: 10.14 (3.75) mins, range: 3–23 min). There was also minimal variation in the day-to-day behavior of individual participants, as evidenced by both the small standard deviations associated with both daily interval count (1.88 intervals/day) and interval duration (5.36 min/day). With respect to myopia status, there was also minimal difference in the frequency of daily outdoor activities between myopes and non-myopes (mean (SD) daily interval count: myopes, 3.26 (1.49); non-myopes, 3.46 (1.58), unpaired t-test *p* = 0.67) (Figure 2). Furthermore, there was no correlation between either the mean daily outdoor interval duration or frequency and either SER or AL (Figure 2, *R*^2^ ≤ 0.05).

**Figure 3:**
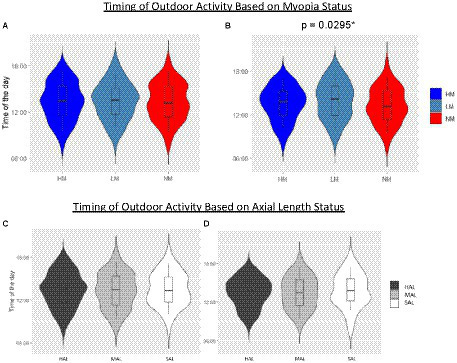
Outdoor activity dynamics. Outdoor activity dynamics defined in terms of the time of day of outdoor activities, shown by myopia and axial length categories for both weekdays [**(A,C)**, respectively] and weekends [**(B,D)**, respectively]. On each violin plot is superimposed a box plot denoting the related median of the distribution and +/− 1SD whiskers.

The myopia status of participants also appeared to have minimal influence on the timing of outdoor activities, at least on weekdays. For both myopic and non-myopic participants, the timing of outdoor activities was quite variable although there was bias towards afternoons, as reflected in the higher percentage of outdoor intervals occurring in the afternoon, i.e., after 12 pm (mean (SD)%: myopes, 68.9 (19.3) %; non-myopes, 73.6 (14.6) %). These behavioral patterns are also reflected in the mean (SD) timing of outdoor activities; 13:22 (2.52 h), 13:27(2.45 h) and 13:20 (2.48 h) for HM, LM, and NM on weekdays, respectively (*p* > 0.05) (Figure 3A). However, on weekends, both HM and LM groups went outdoors slightly later in the afternoon compared to the NM group (mean (SD) time: 13:30 (2.33 h), 13:57(2.58 h) and 13:15 (2.52 h) for HM, LM, and NM on weekends, respectively, *p* = 0.03).

Similar trends were seen when participants were categorized by axial length. Specifically, participants with longer eyes appeared to go outdoors later in the day, compared to those with shorter eyes (Figures 3C,D), although this observation did not reach statistical significance, for either weekdays or weekends. The mean times that HAL, MAL, and SAL groups went outdoors on weekdays were 13:23 (2.47 h), 13:18 (2.53 h), and 13:28 (2 h), respectively and on weekends,13:27 (2.27 h), 13:28 (2.53 h), and 13:48 (2.67 h), respectively.

#### 3.2.3. Outdoor lighting characteristics

**Figure 4:**
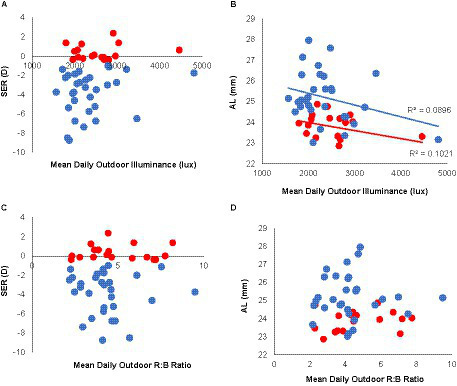
Outdoor lighting characteristics in myopes and non-myopes. Refractive errors (SERs) and axial lengths (ALs), plotted against mean daily illuminance (lux, top panel) and spectral composition expressed as R:B ratios (bottom panel) to which participants were exposed. Daily outdoor illuminance was slightly lower on average for myopes (blue) compared to non-myopes (red) **(A)**. Superimposed solid lines represent results of correlation analyzes. Outdoor illuminance and axial length are weakly correlated **(B)**, while the spectral composition of outdoor lighting shows no correlation in either SER **(C)** or AL **(D)**.

Overall, the light levels (illuminance) to which myopes were exposed in the outdoor environment during APs was slightly lower, on average, than the levels to non-myopes were exposed [mean (SD): 2349.66 (640.69) vs. 2554.70 (607.45) lux], although this difference was not significant (*p* = 0.27), and there was no significant correlation between SER and mean daily outdoor illuminance (Figure 4A). Participants who experienced brighter outdoor lighting also tended to have shorter eyes, although not significantly so (Figure 4B, *p* = 0.24, *R*^2^ ≤ 0.10). The spectral composition of outdoor lighting to which non-myopes compared to myopes were exposed also tended to have a relative bias towards longer wavelengths, as reflected in the relatively higher red radiance values (mean (SD): non-myopes: 5353.68 (1987.81) μW/cm2, myopes: 4209.78 (1763.21) μW/cm2, *p* = 0.05), and the higher ratio of long:short wavelengths (R:B Ratio) (non-myopes: 4.92 (1.87) and myopes: 4.31 (1.58), respectively. However, the R:B ratios were not significantly correlated with either SERs or ALs (Figures 4C,D).

### 3.3. Indoor activity

#### 3.3.1. “Near” behavior

**Figure 5:**
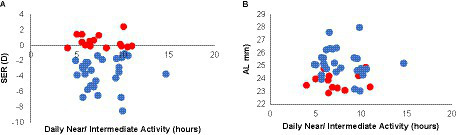
“Near” behavior of myopes and non-myopes. Refractive errors [SERs, **(A)**] and axial lengths [ALs, **(B)**] plotted against mean daily reported “near” activity for myopes (blue) and non-myopes (red); the two groups showed similar and quite variable behavior, which was not significantly correlated with either SER or AL.

During APs, the average amount of daily “near” activity, as indicated by daily Actiwatch inputs by participants, was generally quite variable across participants (range: 4 to 14.7 h, mean (SD): 7.69 (2.22) h). However, in terms of average daily amount of ‘near’ activities performed by myopes versus non-myopes, no significant difference was found (7.82 (2.31) and 7.49 (2.12) h, respectively, *p* = 0.64). There was also no significant relationship between average reported daily ‘near’ activities and either SER or AL (*R*^2^ ≤ 0.08, Figure 5).

#### 3.3.2. Indoor activity dosing

On average, time spent indoors, as measured by the Actiwatch, demonstrated that young adult students spend extended periods of time indoors during APs [mean (SD) count: 12.12 (2.37) intervals/day, range: 6.25 to 17 intervals/day, mean interval duration: 58.31 min, range: 25.35 to 119.77 min]. There was also minimal intra-participant variability in their day-to-day behavior with respect to the number of indoor intervals per day, as reflected in the average standard deviation associated with daily interval count (3.96 intervals/day), although there was more intra-participant variability in the duration of each interval (74.72 min/interval).

**Figure 6:**
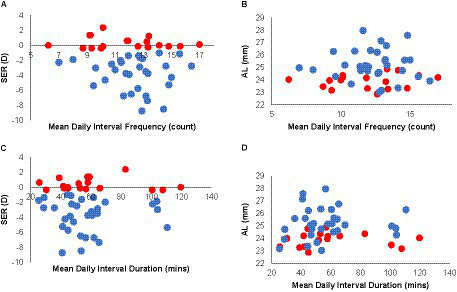
Indoor activity interval dosing in myopes and non-myopes. Refractive errors [SERs, **(A)**] and axial lengths [ALs, **(B)**] plotted against mean daily indoor interval frequency **(A,B)** and interval duration **(C,D)**; trends were both similar for both myopes (blue) and non-myopes (red) and neither variable was significantly correlated with either SER **(A,C)** or AL **(B,D)**.

With respect to myopia status, there was also no significant difference between myopes and non-myopes in the frequency of daily indoor activities, as indexed by both interval counts and durations (mean daily interval count (SD): myopes, 12.36 (2.10); non-myopes, 11.72 (2.79); mean daily interval duration (SD): myopes, 57.27 (21.73) min; non-myopes: 60.09 (26.38) min). Thus predictably, there was no correlation between the mean daily frequency of indoor activity and either SER or AL (Figures 6A,B, R^2^ ≤ 0.06), and likewise, no correlation between mean daily indoor interval duration and either SER or AL (Figures 6C,D, *R*^2^ ≤ 0.07).

#### 3.3.3. Indoor lighting characteristics

**Figure 7:**
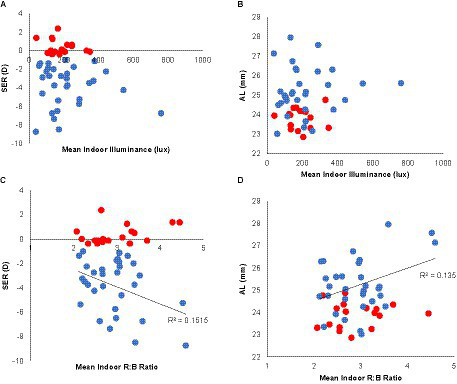
Indoor activity lighting characteristics in myopes and non-myopes. Refractive errors (SERs) and axial lengths (ALs) plotted against mean indoor daily illuminance (lux) **(A,B)**, and spectral composition, as expressed as the R:B ratio **(C,D)**. Compared to non-myopes (red), myopes (blue) experienced slightly higher mean indoor daily illuminance although no significant correlation with SER **(A)** or AL **(B)** was observed. The spectral composition of indoor lighting was similar for non-myopes and myopes, and for myopes, R:B ratios were significantly correlated with both SER [*p* = 0.04, **(C)**] and AL [*p* = 0.03, **(D)**].

Overall, the indoor environments to which myopes were exposed during APs were slightly brighter, albeit more variable compared to those experienced by non-myopes [217.86 (155.90) vs. 182.60 (77.14) lux], and this difference was not statistically significant (unpaired *t*-test, *p* = 0.30). In addition, myopes tended to experience more variation in the brightness of their indoor environments within a day, as reflected in the mean standard deviations in illuminance across indoor activity intervals for a given day (mean: myopes, 337.88 lux, non-myopes, 282.69 lux, *p* = 0.45). Nonetheless, there was no significant correlation between mean indoor illuminance and either AL or SER (Figures 7A,B, *R*^2^ < 0.05).

In general and for all participants, the spectral composition of the indoor environments were cooler compared to their outdoor environments (e.g., proportionally less long wavelength light), as reflected in the reduced R:B ratio [mean (SD) R:B ratio: 2.95 (0.63) and 4.54 (1.70), respectively]. Interestingly, although the indoor experiences, in terms of R:B ratios, of non-myopes and myopes were similar (2.99 (0.67) and 2.93 (0.61), respectively, *p* = 0.77), myopic participants who experienced higher R:B ratios indoors were significantly more myopic and had longer eyes (Figures 7C,D, *R*^2^ = 0.14–0.15, *p* ≤ 0.04).

### 3.4. Influence of academic periods

A comparison of outdoor activity dosing across APs and NAPs for the subset of 14 participants for whom observations were made in both periods revealed a small but statistically significant difference in the frequency of intervals spent outdoors such that all but 3 participants went outdoors more frequently in NAPs compared to APs (1.2 more intervals, on average for *n* = 11, *p* = 0.04). In addition, all but 4 participants went outdoors for slightly, but significantly longer intervals in NAPs (by 2.56 min more on average for *n* = 10, *p* = 0.04). With respect to myopia status, the myopes in this limited sample demonstrated a slightly greater modification of their outdoor behavior than the non-myopes, going outdoors more frequently (1 interval more) and staying outdoors slightly longer (increase of 2.96 min). The latter but not the former difference reached borderline statistical significance (*p* = 0.42 and *p* = 0.07, respectively).

A comparison of the “near” behavior during APs and NAPs in the same subset of 14 participants revealed that they spent significantly less time in “near” activities during NAPs (by on average 1.5 h, *p* = 0.02). For non-myopes and myopes, the reductions in “near” activities during NAPs compared to APs [mean (SD) were 2.09 (1.73) h and 1.27 (2.32) h, respectively]. Thus, on average, non-myopes and, to a lesser extent, myopes, tended to spend less time engaged in “near” activities during NAPs, although these differences were not statistically significant (*p* = 0.49). Interestingly, all non-myopes reported being engaged in less “near” activities in NAPs (range: 0.27 to 3.93 h less), while reports from myopes were more variable, with some reporting more near activities (by 0.27 to 2.50 h) and others less (by 0.56 to 6.28 h) during NAPs.

## 4. Discussion

The study described here made use of an Actiwatch, a wearable light-sensing and activity monitoring device, which has been used to-date, by two other groups to study outdoor behavior in the context of myopia research (4, 14). However, different from these previous studies, we focused on the temporal dynamics of behavior. While much attention has been paid to the possible protective role of outdoors against myopia development, little attention has been paid to the temporal dynamics related to outdoor exposure, and both indoor lighting characteristics and indoor behavior have been largely ignored, even though the proportion of time spent indoors by both children and adults typically far exceeds that spent outdoors. Thus, our analyzes are novel and our study findings have the potential to offer new insights into the inter-relationships between outdoor and indoor exposures/behaviors and myopia.

Overall, for our study population of young adult university students, the dosing of indoor and outdoor activities, as measured in terms of mean daily interval frequencies and durations during an academic period, was not predictive of either myopia presence or amount, or of eye length. While there was a trend linking exposure to increased lighting levels, as encountered outdoors, and shorter axial lengths, no equivalent difference between myopes and non-myopes was observed. There was also no significant difference between these groups in terms of the average frequency and duration of their outdoor activities, although subtle differences in the timing of their outdoor activities were noted. For example, on the weekends myopes appeared to go outside more often later in the day than non-myopes, which is consistent with our finding that they also were exposed to proportionally more long compared to short wavelengths (larger R:B ratios). Interestingly, in relation to indoor activities, longer wavelength light (expressed as an increased R:B ratio) was associated with higher myopia and longer eyes within myopic participants, although the overall illuminance of the indoor environment was not related to either myopia presence or magnitude.

A potential reason for the generally similar indoor and outdoor behaviors in this cohort of students may lie in part to fact that data collection was largely restricted to the academic year, when shared weekday class schedules likely imposed limits on inter-subject variability in behavior. For this reason, we sought to collect data encompassing both academic and non-academic periods in a subset of participants. For this group, we found small but significant increases in the frequency and duration of intervals spent outdoors, as well as reduced near/intermediate work during non-academic periods, with myopes apparently more likely to modify their behavior. The latter observation offers a plausible explanation for independent reports of slower myopia progression in the summer months, which typically corresponds with a non-academic period (28–30). On the other hand, while a number of studies have reported myopia progression in young adult university students (31, 32), our myopic cohort did not show meaningful progression, perhaps reflecting the relatively short duration of our study, i.e., of ~6-months.

Interest in the influence of environmental light levels, and specifically of bright lighting as a potential explanation for the protective effect of outdoors, has been driven largely by results from studies using animal models of myopia. As specific commonly cited examples, bright light rearing conditions were found to protect against form-deprivation myopia in both monkeys (33) and chickens (34, 35). However, for both species, the same conditions offered significantly less protection against lens-induced myopia, a paradigm argued to be more relevant to the most common form of human myopia (36, 37). None-the-less, in chickens, the dosing characteristics (frequency of delivery and brightness) was found to be important determinants of the protective effect against form deprivation myopia, with frequent, very short periods of bright lighting (e.g., 1:1 min cycles), offering better protection than continuous, albeit diurnal exposure to the same lighting (16), for increasing illuminance levels up to 10 klux (38). While these studies provide rationale for investigating outdoor dosing in humans, as undertaken here, methodological differences, including the brightness and spectral composition of the lighting and extended exposure durations (12–14 h), used in these animal studies along with form deprivation to induce myopia in most of them, make it difficult to extrapolate from their conclusions to the human condition. However, given the highly variable lighting conditions to which humans are exposed in daily life, exploration of the dynamics of light exposure would seem a critical step towards advancing our understanding of their influence on human refractive error development and thus myopia, with the methodology described herein being broadly applicable to future observational studies.

To objectively measure light exposure, the advent of commercially available light-sensing, wearable technologies has led to their deployment in an increasing number of myopia-related studies, mostly involving children (2, 4, 14, 39). In general, these studies also found that increased exposure to higher light levels had little to no effect on myopia progression and/or axial elongation in children (e.g., 0.07 mm or less reduction in axial elongation over 6 months to 1 year). Similarly, in the East Asia-based randomized control trials alluded to earlier, increasing outdoor recess time had little to no clinically significant effect on myopia progression (or axial elongation) in already myopic children, although cases of myopia incidence were slightly reduced, by approximately 5–10%. However, in one follow-up study in which participants wore a light sensor device around their necks during the school day (8), the combinations of either longer outdoor intervals (>200 min) and lower illuminance (>1,000 or > 3,000 lux) or slightly shorter intervals (125–199 min) combined with higher illuminance (>10,000 lux) were found to have comparable, albeit small, protective effects against myopia development. Thus, this study offers some support for the monitoring of temporal outdoor exposures dynamics.

Interest in the influence on eye growth regulation of the spectral distribution of lighting has also been driven, at least in part, by investigations involving animal myopia models and the recognition that wavelength influences the refractivity of light, with longer wavelengths coming to focus beyond shorter wavelengths. That young eyes “grow into focus” is also consistent with the growth-enhancing effect of diurnal monochromatic red light, as observed in young chicks, although puzzlingly, the same conditions have the opposite effect, i.e., of slowing growth, in tree shrews and monkeys [for a recent review, see Troilo et al. (40)]. Apart from these apparent species differences, it is also important to note that such diurnal monochromatic light rearing conditions have no comparable ‘human’ environmental experience. Nonetheless, the results reported here suggest a link between more severe myopia and an environmental lighting bias towards longer wavelengths, i.e., higher R:B ratios, in our young adult cohort. The generalizability and potential clinical significance of this finding remains to be addressed, given that much remains to be understood about the influence on human eye growth of the spectral composition of lighting. Equivalent follow-up studies in children, who typically exhibit faster progression of myopia than young adults, are warranted.

That excessive near work represents a significant risk factor for myopia development is a long-held belief, providing the stimulus for decades of mostly human research, focused on various aspects of near work, including but not limited to the amount of near work, habitual working distance and gaze breaks [for a review see Harb et al. (41)]. By tradition, most studies aimed at quantifying near work activities have relied on subjective questionnaires, which are subject to recall and other inherent biases, challenging the significance of the finding of a related meta-analysis that the amount of near work was not strongly related to myopia development in children (18). In the case of children, such data are typically extracted from questionnaires completed by parents, guardians and/or teachers, further calling into question, the reliability of such data. By taking advantage in the current study of one feature of the Actiwatch wearable technology that allows participants to be asked each day to key in the hours of near/intermediate activities performed that day, at a set time (e.g., 1 h before bedtime), we were able to generate a more “objective” measure of time spent in these activities. Based on the derived index of near/intermediate activity, no difference in the behavior of myopes and non-myopes was found, although as previously noted, the academic period of monitoring likely imposed artificial limits on behavior.

Comparison of outdoor activity data collected using both traditional, subjective questionnaires and our more objective Actiwatch-derived method, revealed significant misreporting biases in our university student participants, even when questionnaires covered over a relatively short period of a week. The further suggestion from our data that mis-reporting biases may be differentially affected by myopia status raises concerns about the interpretation of previous studies describing differences between myopes and non-myopes in their subjective reports of indoor and outdoor activities. These biases and inaccuracies are surely compounded in studies of pediatric populations, by the secondary nature of reports from caregivers, as alluded to above.

There are several strengths and limitations to consider in the current study. The main strength is the use of an objective wearable technology to quantify indoor and outdoor lighting exposures and characteristics, including spectral compositions. Pertinent to the current indoor analyzes, that indoor exposure was investigated at all represents a significant strength in of itself. Given the substantial amount of time spent indoors compared to outdoors by all generations, including children, an objective characterization of the dynamics of indoor exposure, including relevant lighting characteristics, is important to our understanding of the human myopia condition. Our approach to data analysis represents another strength; by quantifying exposure in terms of temporal dosing parameters, we were able to capture behavioral dynamics, at the same time, identifying and thus avoiding potential confounders, such as spikes of bright indoor exposures arising from device use or short periods of reduced lighting due to obstruction of the Actiwatch sensor. It is important to note that there are no ‘gold standards’ in defining an outdoor or indoor interval by proxies of illuminance intensity and duration. In fact, the idea that illuminance values greater than 1,000 lux is a good proxy for outdoor activity has been called into question (42). Nonetheless, the dynamic analysis protocols described here can be optimized and applied to other existing or future wearable technologies. Key limitations of our study relate to our participant cohort, who were young adult students; based on their age, slower progression than in children and fewer cases of newly onset myopia are expected, and as already alluded to, student academic life imposes its own limitations. The potential confounding of our results by seasonal disparities between academic and non-academic periods, as well as the small number of participants who were available to participate during a non-academic period represent other limitations. Finally, our study cohort was not balanced by either gender or ethnicity, our participants being predominantly East Asian females, likely due to the skewed demographics of the UC Berkeley School of Optometry student population, who comprised most of our participants. The homogenous nature of our study cohort may also be in part to blame for the lack of significant differences observed in indoor and outdoor behaviors.

Finally, it is important to recognize that the results presented here do not offer any new insights into the development and/or progression of myopia, but rather they highlight a novel, dynamic and more comprehensive way of capturing habitual indoor and outdoor activities, for potential application in future studies. Another, yet to be addressed limitation of all investigations to-date, including the current study, is the failure to acknowledge and capture differences in the visual features of indoor and outdoor environments, which may contribute to the apparent protective effects of outdoor environments, and conversely, myopia-genic effects of indoor. It is imperative that such data be collected, especially in studies involving myopic children. Based on our foundational knowledge about the multi-factorial nature of myopia development, as demonstrated in animal studies, several key aspects of a child’s habitual activities and associated visual environments warrant more attention, including the spectral composition, spatial frequency content/contrast, nearness of objects across the visual field, as well as overall light (illuminance) levels. To-date, distance-sensors attached to spectacle frames have been used to record and monitor near working distances. More comprehensive characterization of the spatial features in 3-D of both indoor and outdoor visual environments is also now within reach, given the advances in imaging technology, as might be integrated into lightweight, wireless, wearable head-mounted devices. Such head-mounted technologies would also have additional merit in measuring the lighting characteristics at eye rather than at wrist level, a current limitation of the research presented here and in previous studies.

## 5. Conclusion and future work

To our knowledge, our study represents the first characterization of the dynamics of habitual outdoor and indoor activities, using data captured from Actiwatches, as an example of wearable technologies. While the findings from our cohort of young adult students did not reveal substantial differences in the behavior of non-myopes versus myopes, or differences related to the amount of myopia, the power of this more objective approach to monitoring behavior should not be understated, given the documented reporting biases detected in completed questionnaires. Together with the increasing availability and ease of adoption of such technologies, there would seem little argument against the wide adoption of the approaches used in this study in future myopia studies, irrespective of the age of participants.

## Data availability statement

The raw data supporting the conclusions of this article will be made available by the authors, without undue reservation.

## Ethics statement

The studies involving humans were approved by UC Berkeley Office of Protected Human Research. The studies were conducted in accordance with the local legislation and institutional requirements. The participants provided their written informed consent to participate in this study.

## Author contributions

EH: Conceptualization, Data curation, Formal analysis, Funding acquisition, Investigation, Methodology, Writing – original draft, Writing – review & editing. ES: Formal analysis, Software, Visualization, Writing – review & editing. CW: Conceptualization, Funding acquisition, Project administration, Supervision, Writing – review & editing.
